# A Kinetic View of Membrane Traffic Pathways Can Transcend the Classical View of Golgi Compartments

**DOI:** 10.3389/fcell.2019.00153

**Published:** 2019-08-06

**Authors:** Areti Pantazopoulou, Benjamin S. Glick

**Affiliations:** Department of Molecular Genetics and Cell Biology, The University of Chicago, Chicago, IL, United States

**Keywords:** Golgi, cisternal maturation, compartments, recycling, COPI, COPII, AP-1, clathrin

## Abstract

A long-standing assumption is that the cisternae of the Golgi apparatus can be grouped into functionally distinct compartments, yet the molecular identities of those compartments have not been clearly described. The concept of a compartmentalized Golgi is challenged by the cisternal maturation model, which postulates that cisternae form *de novo* and then undergo progressive biochemical changes. Cisternal maturation can potentially be reconciled with Golgi compartmentation by defining compartments as discrete kinetic stages in the maturation process. These kinetic stages are distinguished by the traffic pathways that are operating. For example, a major transition occurs when a cisterna stops producing COPI vesicles and begins producing clathrin-coated vesicles. This transition separates one kinetic stage, the “early Golgi,” from a subsequent kinetic stage, the “late Golgi” or “*trans*–Golgi network (TGN).” But multiple traffic pathways drive Golgi maturation, and the periods of operation for different traffic pathways can partially overlap, so there is no simple way to define a full set of Golgi compartments in terms of kinetic stages. Instead, we propose that the focus should be on the series of transitions experienced by a Golgi cisterna as various traffic pathways are switched on and off. These traffic pathways drive changes in resident transmembrane protein composition. Transitions in traffic pathways seem to be the fundamental, conserved determinants of Golgi organization. According to this view, the initial goal is to identify the relevant traffic pathways and place them on the kinetic map of Golgi maturation, and the ultimate goal is to elucidate the logic circuit that switches individual traffic pathways on and off as a cisterna matures.

## The Current Status of the Golgi Apparatus

Cell biologists agree that the Golgi apparatus performs essential functions, but they cannot even draw a diagram of the organelle before uncertainties arise. How many compartments exist within the Golgi, and what do we actually mean by the term “compartment”? How do the different cisternae of the Golgi exchange material, and how are these cisternae connected in space and time? Where and how do resident Golgi proteins localize? Which characteristics are fundamental for the operation of the Golgi, and which ones are particular to certain cell types? Here, we address some of these questions while proposing a conceptual framework to guide ongoing research.

## Roles of the Golgi Apparatus

Several functions are attributed to the Golgi in a range of cell types (Farquhar and Palade, 1981; Mironov and Pavelka, 2008; Wilson et al., 2011). The Golgi receives proteins and lipids from both the endoplasmic reticulum (ER) and the endolysosomal system, and it orchestrates the sorting and distribution of these cargoes. Thus, the Golgi acts as a crossroads in the biosynthetic and endocytic traffic routes. As newly synthesized cargo molecules transit through the Golgi, they meet protein and lipid modifying enzymes, most of which modify glycans. Indeed, owing to its numerous glycosyltransferases and glycosidases, the Golgi is a major carbohydrate factory of the eukaryotic cell, responsible for the biosynthesis of glycosphingolipids, glycoproteins, and extracellular polysaccharides (Mellman and Simons, 1992; Driouich et al., 1993; Stanley, 2011; D’Angelo et al., 2013). These functions are broadly conserved.

Other functions of the Golgi may be restricted to particular organisms. For example, the Golgi has emerged as a signaling hub that coordinates secretion with environmental cues and the cell cycle (Farhan and Rabouille, 2011; Chia et al., 2012; Luini and Parashuraman, 2016). These phenomena are probably specific to animal cells, although some signaling mechanisms might be universally important for regulating Golgi traffic. In vertebrates but not in other organisms, the Golgi promotes the nucleation of microtubules that are important for directional post-Golgi trafficking, cell polarization, and migration (Rios, 2014; Sanders and Kaverina, 2015). In plant and yeast cells but not in mammalian cells, Golgi cisternae serve as early endosomes that receive endocytic cargoes destined for recycling or degradation (Dettmer et al., 2006; Viotti et al., 2010; Day et al., 2018). Perturbation of conserved or organism-specific Golgi processes can result in disease (Zappa et al., 2018).

## Classical Definitions of Golgi Compartments

In most studied cells, Golgi cisternae are arranged in polarized stacks, with a *cis* side that receives traffic from the ER and a *trans* side that delivers secretory cargoes to the plasma membrane and the endolysosomal system (Farquhar and Palade, 1981). The *trans*–most cisterna and its associated tubular projections form the *trans*–Golgi network (TGN) (Klumperman, 2011). Vertebrate cells contain multiple Golgi stacks that are connected laterally by membrane tubules to form the juxtanuclear Golgi ribbon (Klumperman, 2011), but other organisms such as plants, insects, and the yeast *Pichia pastoris* contain individual or paired Golgi stacks that are distributed throughout the cell (Faso et al., 2009; Kondylis and Rabouille, 2009; Papanikou and Glick, 2009). In many fungi such as *Saccharomyces cerevisiae* and *Aspergillus nidulans*, cisternae do not form stacks, and instead are scattered in the cytoplasm (Papanikou and Glick, 2009; Pantazopoulou, 2016). The functional consequences of these differences in Golgi architecture are poorly understood (Lowe, 2011), but an evolutionary analysis indicated that stacking has been lost in multiple eukaryotic lineages and is probably not integral to the operating mechanism of the Golgi (Mowbrey and Dacks, 2009).

For both stacked and non-stacked Golgi organelles, evidence of compartmentation has been obtained. Each cisterna is a membrane-bound compartment in a literal sense, but the term “Golgi compartment” is usually taken to mean a set of functionally equivalent cisternae. Golgi cisternae differ in multiple characteristics including buoyant density (Goldberg and Kornfeld, 1983; Dunphy and Rothman, 1985), morphology (Driouich and Staehelin, 1997; Rambourg and Clermont, 1997), content of specific glycosylation enzymes (Farquhar, 1985; Rabouille et al., 1995; Stanley, 2011), content of specific membrane traffic components (Munro, 2005), membrane thickness (Sharpe et al., 2010), and lipid composition (Bigay and Antonny, 2012; Holthuis and Menon, 2014). Such observations led to an early proposal that the Golgi could be viewed as two organelles in tandem (Rothman, 1981). Later studies suggested that the Golgi could be divided into three compartments that are termed *cis*, medial, and *trans* (Dunphy and Rothman, 1985; Mellman and Simons, 1992). The TGN can be described as a sorting station that produces clathrin-coated vesicles and secretory vesicles at the exit face of the Golgi stack (De Matteis and Luini, 2008), and it is often listed as a fourth Golgi compartment. It was proposed that the yeast Golgi consists of four compartments that might correspond to *cis*, medial, *trans*, and TGN (Brigance et al., 2000). In mammalian cells, an ER-Golgi intermediate compartment (ERGIC) is also present (Appenzeller-Herzog and Hauri, 2006). Golgi compartments have been envisioned as sequential stations in an assembly line (Kleene and Berger, 1993). According to this model, vesicular transport would carry secretory cargoes from one stable Golgi compartment to the next (Glick and Luini, 2011).

A problem with these concepts is that Golgi compartments have not been defined in a precise and general way. For example, morphological distinctions between cisternae are specific to certain cell types (Driouich and Staehelin, 1997; Rambourg and Clermont, 1997). The classification of Golgi compartments is often based on the localizations of glycosylation enzymes, but different glycosylation enzymes show partially overlapping distributions, so the original idea of cleanly separated Golgi compartments is no longer valid (Nilsson et al., 1993; Velasco et al., 1993; Rabouille et al., 1995; Harris and Waters, 1996). Moreover, in the Golgi, glycosylation enzymes and glycan modifications vary substantially between organisms (Munro, 2001). For stacked Golgi organelles, the number of cisternae per stack also varies (Mollenhauer and Morré, 1991), leading to ambiguity about how to group the cisternae into compartments. There are no firm guidelines for assigning a Golgi resident protein to a specific compartment. The vague definitions of Golgi compartments have arguably been more of a hindrance than a help in attempts to understand this organelle.

## Cargo Transport in Maturing Cisternae

The idea of a compartmentalized Golgi fits with the historical assumption that Golgi cisternae are long-lived structures, but many researchers now believe that Golgi cisternae are transient structures that form *de novo*, progressively mature, and then fragment into secretory vesicles and other types of carriers (Glick and Nakano, 2009; Glick and Luini, 2011). This cisternal maturation model is the basis for the discussion that follows. A caveat is that alternative models continue to be put forth, based on data that are seen as being inconsistent with a simple maturation mechanism (Patterson et al., 2008; Pfeffer, 2010; Mironov et al., 2013; Pellett et al., 2013; Dunlop et al., 2017). Moreover, cisternal maturation may be augmented in some cases by specialized Golgi traffic routes, such as intercisternal tubules in mammalian cells (Beznoussenko et al., 2014). Despite such complicating factors, the support for cisternal maturation is strong.

According to the cisternal maturation model, Golgi cisternae turn over on a time scale of minutes. COPII-dependent carriers that emerge from the ER are thought to generate a new cisterna – or in mammalian cells, a new ERGIC element – which recycles transport components to the ER in retrograde COPI vesicles. The cisterna then matures by recycling some of its Golgi proteins to younger cisternae while receiving other Golgi proteins from older cisternae. Intra-Golgi recycling mechanisms include vesicle-mediated traffic of transmembrane proteins, coupled with dissociation and reassociation of peripheral membrane proteins (Munro, 2005; Papanikou et al., 2015). Finally, the cisterna dissolves into secretory carriers. In this scheme, secretory cargoes largely remain within the maturing cisternae.

The original evidence for cisternal maturation came from electron microscopy (Mollenhauer and Morré, 1991). For example, large cargoes such as algal scales can be visualized in Golgi cisternae, which apparently act as forward transport carriers (Becker et al., 1995). The generality of this mechanism was established by a rigorous morphological study of procollagen secretion in mammalian cells (Bonfanti et al., 1998). Cisternal maturation was then directly observed by video fluorescence microscopy of individual Golgi cisternae in *S. cerevisiae* (Losev et al., 2006; Matsuura-Tokita et al., 2006). Two-color imaging revealed that early Golgi proteins depart from a cisterna as late Golgi proteins arrive. Further evidence for Golgi maturation came from studying hyphal cells of *A. nidulans*, in which late Golgi cisternae ultimately dissipate into secretory carriers that move to the growing apex (Pantazopoulou et al., 2014). Recently, three-color imaging of yeast indicated that secretory cargo proteins are continuously present within the maturing cisternae as resident Golgi proteins come and go (Casler et al., 2019; Kurokawa et al., 2019). Based on the similarities between the secretory traffic machineries of fungi and mammals (Duden and Schekman, 1997; Papanikou and Glick, 2009), the maturation pathway seen in fungi is likely to be a conserved feature of the Golgi.

Cisternal maturation is thought to be driven by COPI vesicle-mediated intra-Golgi recycling of resident transmembrane proteins (Schnepf, 1993; Glick and Malhotra, 1998; Pelham, 1998; Rabouille and Klumperman, 2005). COPI vesicles also mediate retrograde traffic from the Golgi (and mammalian ERGIC) to the ER (Szul and Sztul, 2011; Barlowe and Miller, 2013), and some researchers have divided COPI vesicles into two categories: COPIa vesicles that mediate recycling to the ER, and COPIb vesicles that mediate intra-Golgi traffic (Donohoe et al., 2007). The mechanisms that generate two types of COPI vesicles are still unclear, but the distinction is useful, and we will employ the COPIa and COPIb nomenclature here.

Recent work revealed that COPI vesicles are not the only drivers of cisternal maturation. A functional study of yeast indicated that COPI mediates recycling of early but not late Golgi proteins (Papanikou et al., 2015). Intra-Golgi recycling of late Golgi proteins apparently involves clathrin-coated vesicles generated with the aid of the AP-1 adaptor. Yeast AP-1 has long been implicated in the recycling of certain late Golgi proteins (Valdivia et al., 2002; Liu et al., 2008; Spang, 2015), and now yeast AP-1 has been found to be restricted to terminally maturing Golgi cisternae, implying that AP-1 recycles a subset of resident Golgi proteins within this organelle (Day et al., 2018). Interestingly, a secretory cargo protein can also be recycled from older to younger cisternae in an AP-1-dependent manner, suggesting that AP-1 vesicles are capable of transporting diverse contents (Casler et al., 2019). AP-1-dependent retrograde traffic within the secretory pathway exists in mammalian cells as well (Hinners and Tooze, 2003; Hirst et al., 2012; Matsudaira et al., 2015). Therefore, intra-Golgi recycling seems to involve the successive actions of COPI and AP-1 vesicles.

## A Conceptual Framework That Links Cisternal Maturation, Membrane Traffic, and Golgi Transmembrane Protein Localization

Traffic pathways at the Golgi are now broadly characterized, but fundamental questions remain. We still lack a detailed understanding of intra-Golgi recycling. Even more uncertain are the mechanisms that allow resident Golgi proteins to be concentrated in particular sets of cisternae (Banfield, 2011). Finally, as described above, imprecise definitions of compartments have led to ambiguity about the functional subdivisions of the Golgi. We propose that these issues are all related, and that an updated conceptual framework can shed light on long-standing mysteries.

### Concept 1: The Golgi Performs Multiple Functions in an Ordered Way

Golgi stacks have a polarity that reflects progressive changes in the functional properties of the cisternae (Dunphy and Rothman, 1985; Rambourg and Clermont, 1997). This organelle is an intermediate between the ER and the plasma membrane, and a variety of lipid modification reactions – including sphingolipid and phosphoinositide synthesis, sterol traffic, and lipid flipping – transform the biosynthetic membranes of the early secretory pathway (thin, loose lipid packing, low surface charge) to the barrier membranes of the late secretory pathway and plasma membrane (thick, tight lipid packing, negative surface charge) (Bigay and Antonny, 2012; Holthuis and Menon, 2014). Meanwhile, as glycolipids and newly synthesized glycoproteins move through the Golgi, their carbohydrate side chains are modified by a series of glycosylation enzymes (Kornfeld and Kornfeld, 1985). Various glycosylation enzymes are concentrated in different cisternae, and their intra-Golgi distributions tend to reflect their order of action (Shorter and Warren, 2002). An additional order-dependent function of Golgi cisternae is sorting. Early Golgi cisternae produce COPI vesicles that recycle resident transmembrane proteins, while late Golgi cisternae produce several types of carriers that sort proteins and lipids for delivery either to the plasma membrane, or to the endolysosomal system, or to younger Golgi cisternae (Myers and Payne, 2013; Papanikou and Glick, 2014; Day et al., 2018).

By changing the functional properties of a Golgi cisterna over time, the cell acquires options that might not otherwise be available. An example is the sorting of lysosomal hydrolases in mammalian cells (Hasanagic et al., 2015). Glycans on those hydrolases are modified by addition of mannose 6-phosphate sorting tags in the youngest Golgi cisternae, before mannosidases arrive. Another example is the formation of secretory vesicles. Those vesicles are generated from the oldest Golgi cisternae, ensuring that secretory proteins are maximally processed before being delivered to the plasma membrane. The general principle is that the ordered pathway of cisternal maturation allows the Golgi to be an efficient and flexible machine for processing and sorting. Because different organisms harness these capabilities in myriad ways, the Golgi has been described as a “factory for evolvability” (Shorter and Warren, 2002).

### Concept 2: Various Membrane Traffic Pathways Operate at Different Times During Cisternal Maturation

We propose that to characterize Golgi organization, the emphasis should be not on glycosylation enzymes, which differ between organisms, but rather on membrane traffic components, which show conserved distributions in the Golgi. In mammalian and plant cells, the TGN produces clathrin-coated vesicles while earlier cisternae in the stack produce COPI-coated vesicles (Mogelsvang et al., 2004; Staehelin and Kang, 2008). Similarly, in the dispersed fungal Golgi, clathrin labels late cisternae while COPI labels early cisternae (Papanikou et al., 2015; Kim et al., 2016; Schultzhaus et al., 2017; Hernández-González et al., 2019). Another example of differential localization is the Arf1 guanine nucleotide exchange factors (GEFs): the GBF/Gea family acts mainly at the early Golgi, while the BIG/Sec7 family acts at the late Golgi or TGN (Gillingham and Munro, 2007). In yeast, the Rab proteins Ypt1 and Ypt31/32 mark the early and late Golgi, respectively (Kim et al., 2016). A general rule is that any given traffic component operates at a specific time during Golgi maturation.

**FIGURE 1:**
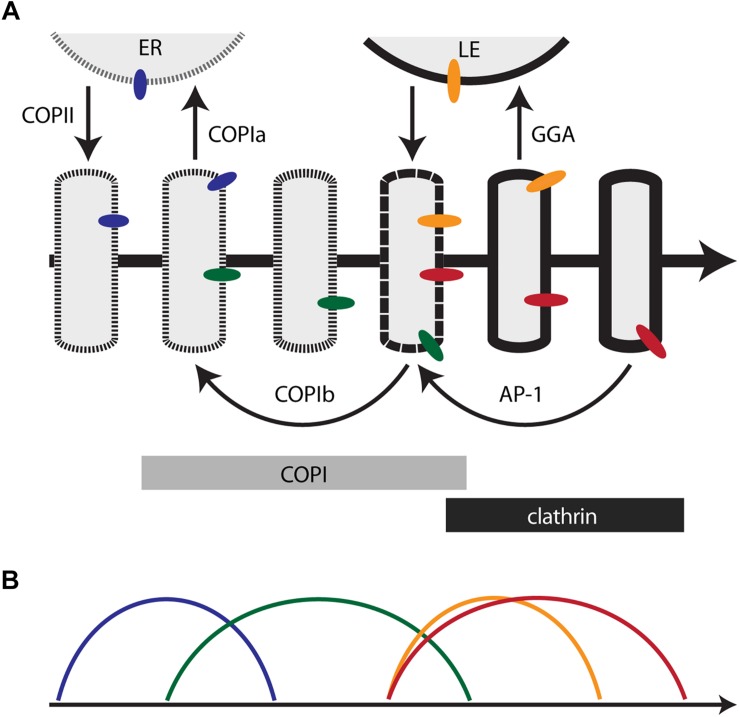
Membrane traffic and the localization of resident transmembrane proteins in the maturing Golgi. **(A)** Diagram of the core membrane traffic pathways that operate at the Golgi. The thick arrow represents the time course of maturation. An individual cisterna evolves along the time axis. In a stacked Golgi, the *cis*-to-*trans* spatial axis would also map onto the time axis. The border of the cisterna changes with time to reflect progressive changes in the lipid bilayer. Thin arrows represent vesicular transport pathways, with relevant coats or adaptors labeled. Bars indicate the approximate residence times for COPI and clathrin on the maturing cisterna. Colored ovals represent Golgi transmembrane proteins that follow different recycling pathways. ER, endoplasmic reticulum; LE, late endosome. **(B)** Predicted kinetic signatures for four different classes of Golgi transmembrane proteins. The colors correspond to those of the colored ovals in **(A)**. See the text for details.

This perspective suggests a possible way to reconcile the ideas of Golgi compartmentation and cisternal maturation. In the maturation model, Golgi compartments could be defined as sequential kinetic stages in the maturation process (Day et al., 2013; Papanikou and Glick, 2014). The relevance of this approach is illustrated by considering the TGN. As traditionally defined, the TGN differs from early Golgi cisternae in prominent ways – it mediates the sorting of biosynthetic cargoes into transport carriers (Griffiths and Simons, 1986; De Matteis and Luini, 2008), it remains separate from the ER when mammalian cells are treated with brefeldin A (Chege and Pfeffer, 1990; Lippincott-Schwartz et al., 1991; Wood et al., 1991), and it often peels off partially or completely from a stacked Golgi (Mollenhauer and Morré, 1991; Mogelsvang et al., 2003; Uemura and Nakano, 2013). When these properties are seen through the lens of Golgi dynamics and membrane traffic pathways, cisternal maturation can be divided into an early stage when the cisternae produce COPI vesicles, and a late or TGN stage when the cisternae produce clathrin-coated vesicles (Figure 1A). This distinction is valuable.

However, the behavior of additional traffic components would require these two stages to be divided further. After Sec7 recruits Arf1 to the late Golgi in yeast, the GGA clathrin adaptor arrives significantly before the AP-1 clathrin adaptor (Daboussi et al., 2012; Day et al., 2018). In *A. nidulans*, the GEF for the Ypt31/32 homolog RabE arrives just before the Sec7-labeled late Golgi dissipates into secretory carriers (Pantazopoulou et al., 2014; Pinar et al., 2015). In the yeast Golgi, the Tlg1 SNARE arrives and departs somewhat earlier than Sec7 (Day et al., 2018), and the Golgi residence time of the Rab protein Ypt6 partially overlaps with the residence times of Ypt1 and Ypt31/32 (Suda et al., 2013). Similarly, certain mammalian membrane traffic proteins are concentrated in medial/*trans* cisternae rather than being restricted to the early or late Golgi (Volchuk et al., 2004; Liu and Storrie, 2012). Morphological analysis of plant cells implied that the early Golgi first undergoes an assembly phase in which COPII vesicles are received while COPIa vesicles are produced, and then undergoes a biosynthetic phase in which COPIb vesicles are received and produced (Staehelin and Kang, 2008). Attempts to assign all of these events to discrete kinetic stages become increasingly contrived. We conclude that it would be unproductive to enumerate all of the kinetic stages of maturation in an effort to define a full set of Golgi compartments.

### Concept 3: Golgi Traffic Pathways Undergo Switch-Like Transitions During Cisternal Maturation

As an alternative to classifying Golgi compartments, we propose that the Golgi should be viewed as a maturing structure controlled by a logic circuit that turns various membrane traffic pathways on and off at different times. During cisternal maturation, each traffic pathway has either a membrane import activity that must be switched on and off at the Golgi, or a membrane export activity that must be switched on and off at the Golgi, or both activities in the case of intra-Golgi recycling. Certain traffic pathways are connected by functional links. These links can be direct – e.g., if a GTPase for one traffic pathway recruits an activator or deactivator of a GTPase for another traffic pathway – or indirect – e.g., if an intra-Golgi traffic pathway recycles an activator of an earlier traffic pathway. Maturation is driven by the traffic pathways. Because the Golgi is a flow system that exchanges membrane both internally and with other organelles, a constraint on the logic circuit is that it must maintain the homeostasis of the endomembrane system.

There is evidence that transitions between Golgi traffic pathways are rapid. A pioneering electron tomography study of the mammalian Golgi indicated that clathrin-coated vesicles bud exclusively from the *trans*–most cisterna while COPI vesicles bud exclusively from earlier cisternae (Ladinsky et al., 1999). Subsequent kinetic analyses of yeast cisternal maturation revealed abrupt changes in the levels of Golgi resident proteins (Losev et al., 2006; Papanikou et al., 2015; Day et al., 2018). We suggest that the transitions that turn each Golgi traffic pathway on and off are switch-like, triggered when the cisterna crosses a threshold for activation or deactivation. During the intervals between transitions, “micro-maturation” may occur in the form of smaller-scale changes in cisternal properties such as membrane lipid composition (Day et al., 2013).

In the Golgi logic circuit, the order of transitions is expected to be fixed for a given cell type and largely conserved in evolution, but the intervals between transitions might vary. By changing the interval between an “on” transition and the corresponding “off” transition, cells could modulate the number of cisternae in a Golgi stack (Bhave et al., 2014). The implication is that aspects of Golgi architecture can be understood in terms of maturation dynamics.

### Concept 4: Resident Golgi Protein Localization Reflects the Operation of Traffic Pathways

Different resident Golgi proteins are known to be concentrated in distinct sets of cisternae, and some of the signals that confer localization to specific parts of the Golgi have been characterized (Banfield, 2011), but the mechanisms that establish this polarity are obscure. For Golgi peripheral membrane proteins, GTPases are typically involved in membrane recruitment (Munro, 2005). Golgi-localized GTPase systems exhibit crosstalk that can drive sequential association and dissociation of a series of GTPases and their effectors (Segev, 2011; McDonold and Fromme, 2014). For Golgi transmembrane proteins, vesicular traffic pathways determine localization. In the simplest scenario, a given Golgi transmembrane protein arrives at a cisterna when the import activity of a traffic pathway is switched on, and then departs from the cisterna when the export activity of the same or a different traffic pathway is switched on. If two Golgi transmembrane proteins do not follow the same traffic pathway, those two proteins will not always be present at the same time in a maturing cisterna. The traditional view would be that the two proteins reside in different Golgi compartments, whereas the maturation-based view is that the two proteins have different kinetic signatures that reflect their traffic pathways.

According to this updated conceptual framework, the challenge of learning how the Golgi works comes down to the following tasks. First, we need to obtain a robust picture of the traffic pathways that operate at the Golgi. Second, we need to understand how the Golgi logic circuit switches those traffic pathways on and off. Third, we need to determine which traffic pathways are used by particular Golgi proteins. The remainder of our review provides a brief discussion of these tasks.

## Golgi Traffic Pathways

Even though the components that drive vesicle budding, targeting, and fusion in the secretory pathway have been characterized in biochemical and structural detail, the physiological roles of those vesicles are still being debated. Figure 1A depicts a working model for Golgi traffic based on an interpretation of existing knowledge. For clarity, this model incorporates a simplification: the traffic pathways are illustrated as operating sequentially, but in fact some of them probably overlap. Other uncertainties in the model are pointed out below.

### COPII-Mediated ER-to-Golgi Anterograde Traffic

New Golgi cisternae form through COPII-dependent export from ER exit sites (ERES) (Barlowe and Miller, 2013). In fungi and plants, the ER export pathway seems to involve spherical COPII vesicles, which fuse homotypically to make a Golgi cisterna (Mogelsvang et al., 2003; Staehelin and Kang, 2008). In animal cells, the ER export pathway is less well defined and may involve COPII-dependent extrusion of large membrane carriers (Raote and Malhotra, 2019). Regardless of the detailed mechanism, secretory cargo proteins are exported in a COPII-dependent manner from the ER and are encapsulated in a newly assembled Golgi cisterna.

### COPIa-Mediated Golgi-to-ER Retrograde Traffic

The best characterized function of COPI vesicles is to recycle proteins to the ER (Barlowe and Miller, 2013). Recycling occurs mainly from the youngest Golgi cisternae (or the mammalian ERGIC) (Klumperman, 2011). In fungal and plant cells, COPI vesicles have been visualized at the interface between ERES and the first Golgi cisterna (Mogelsvang et al., 2003; Staehelin and Kang, 2008). The location of these COPI vesicles, referred to here as COPIa vesicles, suggests that they correspond to the Golgi-to-ER retrograde carriers that have been characterized functionally.

A number of proteins are recycled in a manner suggesting that they are transported in COPIa vesicles. These recycling proteins include soluble ER proteins bound to the KDEL/HDEL receptor, transmembrane ER proteins with C-terminal KKxx retrieval signals, transmembrane ER proteins bound to the Rer1 receptor, and the p24 family of transmembrane proteins (Cosson and Letourneur, 1997; Sato et al., 2001; Aguilera-Romero et al., 2008; Bräuer et al., 2019). Although not explicitly shown in Figure 1A, COPII vesicles might continue to fuse with a newly assembled cisterna after COPIa vesicles begin to recycle proteins to the ER.

### COPIb-Mediated Intra-Golgi Retrograde Traffic

COPI vesicles were initially discovered as intra-Golgi carriers (Orci et al., 1986; Malhotra et al., 1989; Rothman and Wieland, 1996), but the details of this pathway are still murky. Intra-Golgi COPI vesicles, referred to here as COPIb vesicles, are often abundantly present in the vicinity of Golgi cisternae (Ladinsky et al., 1999; Klumperman, 2011). COPIb vesicles have been proposed to travel in the anterograde direction or the retrograde direction or both, or to percolate non-directionally, and they have been proposed to carry either secretory cargo proteins or resident Golgi proteins or SNARE proteins (Rothman and Wieland, 1996; Orci et al., 2000; Rabouille and Klumperman, 2005; Fusella et al., 2013; Pellett et al., 2013). Our assumption is that COPIb vesicles function mainly to recycle Golgi transmembrane proteins from older to younger cisternae (Papanikou and Glick, 2014).

Figure 1A depicts a hypothetical pathway for COPIb vesicles. They are postulated to bud while a cisterna undergoes the changes that will lead to clathrin recruitment. Budding might be triggered by the arrival of COPI-interacting factors that are not present earlier in the maturation process (Witkos et al., 2019; Zhao et al., 2019). After budding, COPIb vesicles are postulated to fuse with younger cisternae. This fusion event probably occurs after cisternal assembly, as suggested by evidence that glycosylation enzymes are not yet active in the earliest Golgi cisternae (Donohoe et al., 2013; Casler et al., 2019). A consequence of the scheme shown in Figure 1A is that between the times of COPIa vesicle budding and COPIb vesicle budding, a cisterna may experience an interval when traffic is minimal. This interval would allow for glycosylation and lipid metabolism, and it might be prolonged in cell types that produce elaborate carbohydrate structures (Glick and Malhotra, 1998). We emphasize that while the model illustrated in Figure 1A is appealing, the COPIb vesicle pathway is still incompletely characterized, and other membrane flow patterns could support intra-Golgi recycling (Rabouille and Klumperman, 2005; Day et al., 2013).

Presumably, COPIb vesicles differ from COPIa vesicles with regard to both the cargoes that are packaged and the specificity of vesicle targeting. COPIa and COPIb vesicles were defined by morphological criteria, based on the locations and staining intensities of vesicles visualized by electron microscopy in plant and algal cells (Donohoe et al., 2007; Staehelin and Kang, 2008). Additional morphological studies of mammalian cells suggested the existence of two populations of COPI vesicles (Orci et al., 1997, 2000). This distinction evidently does not reflect differences in the subunit composition or structure of the COPI coat (Bykov et al., 2017; Adolf et al., 2019). Instead, biochemical studies have suggested that COPI vesicles can package two alternative classes of cargo, together with two alternative sets of vesicle tethers (Lanoix et al., 2001; Malsam et al., 2005). A possible explanation is that a single COPI machinery generates two types of vesicles based on the protein and lipid compositions of the parental cisternae.

### Bidirectional Traffic Between the Golgi and Late Endosomes

Traffic of acid hydrolases from the Golgi to late endosomes (or yeast prevacuolar endosomes) is mediated by sorting receptors that are recognized by the GGA clathrin adaptors (Bonifacino, 2004; Hirst et al., 2012; Myers and Payne, 2013). Kinetic studies of yeast cells indicated that GGA is recruited as soon as Sec7 and clathrin arrive, and significantly before the final phase of cisternal maturation (Daboussi et al., 2012). Therefore, Figure 1A depicts GGA-dependent traffic to late endosomes as taking place before the formation of secretory vesicles.

Acid hydrolase sorting receptors recycle from late endosomes to the Golgi by pathways that involve sorting nexins (Chi et al., 2015; Wang et al., 2018). These sorting receptors must be returned to the Golgi to bind acid hydrolases prior to GGA recruitment, so recycling carriers from late endosomes are depicted as arriving shortly before the COPI-to-clathrin transition.

### AP-1-Mediated Intra-Golgi Retrograde Traffic

The functions of AP-1 have long been a puzzle, but as described above, evidence from mammalian and yeast cells indicates that an important role of AP-1 is to mediate retrograde traffic in the late secretory pathway (Bonifacino, 2014; Spang, 2015; Day et al., 2018). Yeast AP-1 is recruited to Golgi cisternae after GGA, and persists longer than any other characterized Golgi marker (Daboussi et al., 2012; Day et al., 2018; Casler et al., 2019). AP-1 is inferred to mediate intra-Golgi recycling during a late phase of cisternal maturation. Based on kinetic studies of the recycling of Golgi transmembrane proteins and of a secretory cargo in yeast, Figure 1A depicts AP-1 vesicles as arriving at about the same time that COPI is being replaced with clathrin.

Even though AP-1 arrives and departs after GGA, the residence times of these two adaptors overlap (Daboussi et al., 2012). Thus, GGA might continue to transport acid hydrolases after AP-1 has begun to recycle Golgi transmembrane proteins.

### Additional Golgi Traffic Pathways

Golgi traffic pathways beyond those already listed have been described. For example, in yeast cells, endocytic vesicles are targeted directly to the Golgi and arrive just before Sec7 (Day et al., 2018). The AP-3 adaptor is responsible for delivering certain newly synthesized transmembrane proteins to the lysosome or vacuole (Odorizzi et al., 1998; Llinares et al., 2015), and in the yeast Golgi, AP-3 probably operates soon after Sec7 arrives (Day et al., 2018). Although these pathways are undoubtedly important, we propose that Figure 1A is an adequate working model for the conserved core traffic machinery of the Golgi.

## Control of Golgi Traffic Pathways

To understand how Golgi traffic pathways are controlled – i.e., to describe the underlying logic circuit – we need to determine which components act in a given traffic pathway. Most of the relevant components are probably known. In addition to the coats and adaptors that have been mentioned, other players are crucial. An in-depth treatment is beyond the scope of this review, but we will highlight some of the key proteins.

• GTPases regulate multiple aspects of Golgi traffic (Mizuno-Yamasaki et al., 2012). During cisternal maturation in yeast, the Rab proteins Ypt1, Ypt6, and Ypt31/32 are activated sequentially and have partially overlapping residence times (Suda et al., 2013; Kim et al., 2016). The Arl1 GTPase acts at about the same time as Ypt6 (Yu and Lee, 2017).
• Tethering proteins capture vesicles arriving at the Golgi (Witkos and Lowe, 2017). A functional study classified Golgi tethers into three categories based on the types of cargo-containing vesicles that were captured (Wong and Munro, 2014; Gillingham and Munro, 2016). This tether classification scheme can be provisionally integrated with Figure 1A, as follows. (1) The first class of tethers operates at newly assembled cisternae to capture COPII vesicles as well as COPIb vesicles. (2) The second class of tethers captures vesicles arriving at the Golgi from late endosomes. (3) The third class of tethers captures intra-Golgi AP-1 vesicles. A test of this interpretation will require further molecular analysis of the vesicle types recognized by the different tethers.
• SNARE proteins drive vesicle fusion, and also contribute to vesicle targeting specificity (Malsam and Söllner, 2011). Several SNARE complexes operate within the Golgi. The SNARE complex that mediates fusion of COPII vesicles is functionally well characterized (Liu and Barlowe, 2002), but less is known about the SNAREs and SNARE complexes that mediate the other three pathways shown in Figure 1A for incoming vesicle fusion at the Golgi.

To fill in missing pieces of the membrane traffic story, it will be essential to determine when each traffic component arrives and departs during cisternal maturation. Such kinetic mapping can be performed by video confocal microscopy of tagged yeast Golgi proteins (Losev et al., 2006; Matsuura-Tokita et al., 2006; Papanikou et al., 2015; Ishii et al., 2016; Day et al., 2018). An analogous approach for mammalian cells is high-resolution spatial mapping of resident proteins in Golgi stacks (Tie et al., 2016). Although these sorts of mapping studies cannot prove a particular mechanism, they can constrain possible mechanisms.

Perhaps the most interesting challenge is to elucidate the functional links that define the Golgi logic circuit. Maturation can arise readily in vesicular transport systems, but multiple traffic networks are possible (Mani and Thattai, 2016). An individual traffic pathway is presumably switched on and off through the actions of other traffic pathways. Insight into these functional links is gradually emerging. We will highlight a few examples:

• Rab GTPases are thought to operate in cascades during organelle maturation (Rivera-Molina and Novick, 2009; Mizuno-Yamasaki et al., 2012). In this scenario, an early acting Rab activates a GEF that promotes association of a late acting Rab, which in turn activates a GTPase activating protein (GAP) that promotes dissociation of the early acting Rab. For example, Ypt32 activates a GAP for Ypt6, which acts earlier during Golgi maturation (Suda et al., 2013).
• GGA promotes the activation of a phosphatidylinositol 4-phosphate (PI4P) kinase, and PI4P in turn is needed to recruit AP-1 (Daboussi et al., 2012, 2017).
• The peripheral membrane protein Sec7 is recruited through the action of multiple GTPases, including Ypt1 and Arl1, which become active earlier in the maturation pathway (McDonold and Fromme, 2014).
• The COG vesicle tether interacts with multiple components that operate in different Golgi traffic pathways (Willett et al., 2013), suggesting that one of its roles may be to connect the AP-1-dependent and COPIb-dependent pathways for intra-Golgi recycling.

Based on Figure 1A, we can speculate about additional functional links. For example, the yeast Vps74 protein has been implicated in COPIb-dependent recycling of resident Golgi proteins, but Vps74 recruitment requires PI4P, which is synthesized late in maturation after GGA arrival (Tu et al., 2008; Graham and Burd, 2011; Daboussi et al., 2012). It seems plausible that AP-1-dependent recycling delivers PI4P to younger cisternae, thereby helping to trigger COPIb vesicle budding. This idea should be experimentally testable. In general, functional links between Golgi traffic pathways can be revealed by examining how targeted perturbations alter Golgi maturation.

## Traffic-Dependent Localization of Golgi Transmembrane Proteins

In both stacked and non-stacked Golgi organelles, resident transmembrane proteins are concentrated in different parts of the Golgi. We propose that this phenomenon reflects the multiple traffic pathways that mediate Golgi recycling. In Figure 1A, the blue, green, orange, and red ovals represent Golgi transmembrane proteins, and in Figure 1B, the colored curves depict the corresponding kinetics of arrival and departure for each protein. The model predicts the following:

• The blue protein recycles through the ER via COPIa vesicles. As a result, this protein is present in both the ER and the youngest Golgi cisternae (including the ERGIC in mammalian cells). Candidates for transmembrane proteins that follow this pathway include Rer1 and the p24 proteins (Sato et al., 2001; Strating and Martens, 2009).
• The green protein recycles within the Golgi via COPIb vesicles. As a result, this protein arrives soon after the blue protein, and departs by the time that clathrin appears. A candidate for a transmembrane protein that follows this pathway is the yeast GDP-mannose transporter Vrg4 (Abe et al., 2004; Losev et al., 2006; Papanikou et al., 2015). Other glycosylation enzymes of the early Golgi probably follow a similar pathway. Consistent with this interpretation, accumulating evidence indicates that certain mammalian glycosylation enzymes can be packaged into COPI vesicles (Liu et al., 2018; Adolf et al., 2019).
• The orange protein recycles between the Golgi and late endosomes. As a result, this protein arrives before clathrin appears, and departs soon after clathrin appears. A candidate for a transmembrane protein that follows this pathway is the yeast acid hydrolase receptor Vps10 (Cooper and Stevens, 1996).
• The red protein recycles within the Golgi via AP-1 vesicles. As a result, this protein arrives soon before clathrin appears, and departs during the terminal phase of Golgi maturation. A candidate for a transmembrane protein that follows this pathway is the yeast SNARE Tlg1 (Valdivia et al., 2002; Day et al., 2018). It is also possible that some late acting glycosylation enzymes, such as yeast Mnn1 and mammalian β1,4-galactosyltransferase 1 (Graham et al., 1994; Schaub et al., 2006), recycle in AP-1 vesicles from the late Golgi/TGN to earlier cisternae.

Some transmembrane Golgi proteins may follow more complex itineraries, in which case they will not fit into any of these four categories. For example, the yeast SNARE Sed5 – homologous to mammalian syntaxin-5 – cycles through the ER, presumably via COPIa vesicles, but is also present and active later in the Golgi, suggesting that a subset of the Sed5 molecules recycle via COPIb vesicles (Wooding and Pelham, 1998; Kurokawa et al., 2019). Moreover, the mammalian cation-independent mannose 6-phosphate receptor (CI-MPR) travels to late endosomes in GGA vesicles (Bonifacino, 2004), but is also present in AP-1 vesicles and in cisternae near the *trans* face of the stack (Doray et al., 2002; Tie et al., 2016), suggesting that a subset of the CI-MPR molecules recycle within the Golgi via AP-1 vesicles. Despite these subtleties, the long-standing problem of determining the localization mechanism for a Golgi transmembrane protein can potentially be solved by describing the protein’s traffic itinerary.

## Concluding Remarks

A vast amount of detailed information has been gathered about elements of the Golgi machine, but the field has lacked a compelling framework for integrating this information. Here, we present a new attempt to construct such a framework. Cisternal maturation is proposed to be controlled by a logic circuit that switches a series of conserved traffic pathways on and off in a choreographed manner. This hypothesis has the virtue of making testable predictions that will advance our understanding. Sooner or later, the Golgi will give up its secrets.

## Author Contributions

The ideas were developed through a dialogue between AP and BG. AP wrote the initial draft of the manuscript and generated the figure. BG revised the manuscript.

## Conflict of Interest Statement

The authors declare that the research was conducted in the absence of any commercial or financial relationships that could be construed as a potential conflict of interest.
